# Gut microbiome modulation mediated by probiotics: Positive impact on growth and health status of *Labeo rohita*

**DOI:** 10.3389/fphys.2022.949559

**Published:** 2022-09-09

**Authors:** Ifra Ghori, Misbah Tubassam, Tanveer Ahmad, Amina Zuberi, Muhammad Imran

**Affiliations:** ^1^ Department of Microbiology, Faculty of Biological Sciences, Quaid-i-Azam University, Islamabad, Pakistan; ^2^ Department of Biotechnology, Fatima Jinnah Women University, Rawalpindi, Pakistan; ^3^ Fisheries and Aquaculture Laboratory, Department of Animal Sciences, Quaid-i-Azam University, Islamabad, Pakistan

**Keywords:** microbiome, metagenomics, *Labeo rohita*, probiotics, physiology, feed utilization capacity

## Abstract

The current study was targeted to determine the effect of probiotics on the growth, physiology, and gut microbiology of *Labeo rohita* fingerlings. One hundred and twenty fishes were divided into four dietary groups, each in triplicate for a feeding trial of 90 days. These treatments included T0 (control, basal diet) used as the reference, and three probiotic-supplemented diets represented as Tbc (*Bacillus cereus*), Tgc (*Geotrichum candidum*), and Tmc (*B. cereus* and *G. candidum*). The probiotics were supplemented at a level of 1 × 10^9^ CFU/g feed. Fishes nurtured on probiotic-added diet showed significantly high physiological improvement (*p* < 0.05) in terms of growth, feed utilization capacity, hematological profile, and digestive enzymes as compared to control. The fish were subjected to a challenge test after a 90-day feeding trial. The Tmc exhibited maximum fish growth when challenged by *Staphylococcus aureus* and showed fish survival when compared to control, in which fish mortality was examined. Fish gut microbial composition was modulated by probiotic treatments, especially in Tgc and Tmc as compared to control. The absence of opportunistic pathogens such as *Staphylococcus saprophyticus* and *Sporobolomyces lactosus* and detection of lower levels of *Trichosporon* and *Cryptococcus* in treated groups indicate the gut modulation driven by applied probiotics. The *G. candidum* QAUGC01 was retrieved in yeast metagenomics data, which might be due to the production of polyamines by them that facilitated adherence and consequent persistence. In conclusion, it can be suggested that the probiotic-supplemented diet could enhance fish growth and feed efficiency through community modulation and digestive enzymes, which could be a milestone in local aquaculture.

## 1 Introduction

The aquaculture industry has a leading role in the accomplishment of an increasing demand for high-quality animal protein. Poor feed conversion ratio and high mortality hinder fish growth, thus resulting in decreased fish production. The practice of antibiotic treatment in aquaculture is bound with the evolution of resistant bacterial pathogens in humans (Allameh et al., 2016). An upsurge in antibiotic resistance and consumer safety concerns are key drivers that motivated scientists to seek alternate control strategies (Pandiyan et al., 2013). Probiotics as dietary additives are an alternative pathogen control strategy instead of antibiotic treatments (Bandyopadhyay et al., 2015; Wu et al., 2015; Kuebutornye et al., 2019). Gut microbiota is associated with gut development, immunity, and metabolism of the host (Pérez et al., 2010; Nicholson et al., 2012; O’shea et al., 2012; Butel, 2014; Koch et al., 2018). Imbalanced fish gut microbiota leads to poor metabolism, reduced growth, stress, and disease onset. The overall impact of gut microbiota on the physiology of fishes is dependent on the composition of gut microbial communities (Vigneri, 2014). Gut microbiota is primarily established by diet (Miyake et al., 2015). The detailed knowledge of the bacterial community through next-generation sequencing is instrumental in target modulation of bacterial communities for better fish health and productivity (Wang et al., 2018; Ou et al., 2021). Fish gut microbiota can be manipulated through prebiotics, antibiotics, and probiotics to investigate the role of gut microbiota (Butt and Volkoff, 2019). Novel dietary ingredients, especially prebiotics, probiotics, and synbiotics as feed supplements, are regulatory factors of physiology, growth, and health. Due to their impact on the gut microbiome, they are receiving the attention of researchers as fish feed additives (Kuebutornye et al., 2019; Tacon and Metian, 2008; Miyake et al., 2015; Butt and Volkoff, 2019). The presence of similar gut core microbiota from geographically and taxonomically distinct fishes is indicative of their functions as metabolism and physiology regulatory agents (Talwar et al., 2018).

Evidenced-based research has validated the link between probiotics and improved food efficiency and overall physiology. Furthermore, host-originated probiotics could be the best strategy for sustainable aquaculture (Zorriehzahra et al., 2016). Native fish gut microbes are not only cost-effective but also have immense digestive enzyme potential (Ganguly et al., 2010). Commercially available feed supplemented with probiotics has shown a promising increase in growth and feed conversion efficiency in fish and shellfish (Gumus et al., 2016; Mohammadi et al., 2016). Probiotics protect the host by several modes: excluding pathogens by competing for attachment sites or production of antimicrobials, providing nutrients, and acting as growth promoters or exo-enzymes and thus helping in digestion, water quality management, stimulation of hematopoiesis, and immune stimulation (Mukherjee et al., 2019; Munir et al., 2018; Nandi et al., 2018).

In comparison, a combination of probiotics enhances overall efficiency in growth, survival, and immunity. Digestive enzyme titers are more in the combination of probiotics than in monoculture (Sun et al., 2010; Mohapatra et al., 2012; Ringo et al., 2016). *Labeo rohita* is grown/cultivated at a large scale in Asia due to its high commercial demand and consumer preference. *L. rohita* can easily succumb to stress, has low feed conversion efficiency, and can suffer from infectious diseases in aquaculture (Choudhury et al., 2005). Because of these mentioned reasons, *L. rohita* was selected for current research. Most of these studies reported the normal gut bacterial community modulation caused by the diet having different sources of protein, while a few studies reported that modulation in both bacterial and fungal communities was caused by probiotic supplementation in the diet.

The principal focus of the conducted study was to investigate the changes in intestinal microbial communities by supplementation of different probiotic treatments in feed, and second, to assess the impact of these treatments in terms of growth, hematology, and other physiological parameters as compared to fish fed on a basal diet. The aquaculture industry recognizes the need for alternatives to chemotherapeutics, antibiotics, and additives to supplement conventional fish feed which will be comparatively beneficial to farmed fish. It was hypothesized that probiotic supplementation would modulate the gut microbiome of *L. rohita* and improve its growth and health status.

## 2 Materials and methods

### 2.1 Sampling of *Labeo rohita*

One hundred and twenty fingerlings of *Labeo rohita*, with an average body weight of 5.90 ± 0.45 were purchased from the Govt. Faisalabad Fish Hatchery, Faisalabad, Pakistan (FFH). The fingerlings were shifted to a fiberglass tank with continuously aeration and free-flowing de-chlorinated water. Fish fed with a basal diet containing 35% protein at a rate of 3% body weight twice a day (8:00 a.m. and 4 p.m.) for 2 weeks at a temperature of 25 ± 1°C were acclimatized to laboratory conditions before the feeding trial. The current study was ethically approved by the Bio-Ethical Committee (BEC) of Quaid-I-Azam University, Islamabad, Pakistan, under protocol number #BEC-FBS-QAU218-115. All experiments were conducted as per International Animal Research Regulations.

### 2.2 Isolation and preparation of probiotics

Previously isolated potential probiotics *Geotrichum candidum* (QAUGC01, NCBI accession number: KT280407) isolated from local fermented milk product curd and *Bacillus cereus* (QAUBC02, NCBI accession number: KY450763) isolated from the gastrointestinal tract of *Labeo rohita* were used. Probiotics both in the form of single-use and combination were used. The synergistic effect on the consortium was checked in *in vitro* conditions prior to their application in the feeding trial. *G. candidum* having GRAS status is FDA-approved, while *Bacillus cereus* was isolated from the fish gut (Kavitha et al., 2018; Das et al., 2021). The bacterial culture was grown on tryptone soya agar and broth (TSA & TSB Oxoid, Basingstoke, Great Britain) at 37°C for 24 h, while yeast was grown on oxytetracycline glucose agar and broth (OGA & OGB; Oxoid Basingstoke, Great Britain) at 30°C for 48 h in a static and shaker incubator (New Brunswick™ Innova^®^ 43 and IRMECO KB 53 UL (E3.1) incubator). Culture stocks were preserved in sterile TSB and OGB containing glycerol v/v 30% and stored at −20°C and −70°C, respectively. Probiotic characterization, antibiotic sensitivity, and antimicrobial activity of the probiotics are presented in Supplementary Tables S1, S2, and S3.

### 2.3 Feed preparation and top dressing

Finely ground dried feed ingredients (Table 1) and vegetable oil were mixed in a fixed proportion, blended, and the dough was kneaded by adding sterile distilled water to prepare a basal diet containing 35% protein. The thin strands of feed were prepared and dried at low temperature in sterile conditions. Afterward, pellets were formed by cutting them into small pieces. The probiotic was dissolved in phosphate buffer pH 7.4, 0.1M and then added to the feed by spraying under sterilized condition in a laminar flow hood. Afterward, the feed was transferred to sterile zipper bags and shifted to the refrigerator. The feed was then supplemented by a pure culture of *G. candidum* QAUGC01 and *Bacillus cereus* QAUBC02 at a concentration of 1 × 10^9^ CFU/g by spraying on a basal diet containing 35% protein in a biosafety cabinet, while the control diet was sprayed with saline and dried at room temperature and stored at 4°C to maintain cell viable count, and it was checked on a weekly basis for its viability and shelf life. It is a documented fact that probiotics in the range of 10^8^–10^10^ CFU/ml are very effective in immune system improvement, regulation of gut microflora, reduction in systems of lactose intolerance, and finally in the prevention and elimination of gastrointestinal infections (Boylston et al., 2004; Zheng et al., 2017). Hence, the probiotic concentration selected for the present study was 1 × 10^9−^Cfu/g. The feed was prepared every 2 weeks to ensure the desired probiotic level. Randomly, probiotic enumeration was also carried out to ensure desirable probiotic concentration.

**TABLE 1:** Recipe 35 percent protein basal diet for *Labeo rohita* (vitamin premix contains vitamins, amino acids, and minerals premix kg-1).

Ingredient	Amount (g/kg)
Soybean meal (46.2% CP)	212
Sunflower meal (40% CP)	212
White fish meal (55%CP)	105
Gluten 30% (30% CP)	105
Canola meal (21.3% CP)	212
Rice polish (13.2% CP)	52
Dicalcium phosphate	10
Carboxymethyl cellulose	10
Vitamin premix^a^	20
Vegetable oil	10
Wheat bran	52
Proximate analysis of basal diet	
Protein	35%
Lipids	8%
Ash	8.7%
Crude fiber	6%

a Vitamin premix contains vitamins, amino acids, and minerals premix kg^−1^.

CP, crude protein, manganese USP, 30,000 mg, vitamin AB.P, 40,000,000 IU, vitamin D3B.P, 820,000 IU, vitamin K3B.P, 800 mg, L. lysine B.P, 10,500 mg, vitamin B2B.P, 2,500 mg, vitamin EB.P, 6,200 mg, vitamin B12B.P, 1,000 mg, vitamin B3B.P, 5,100 mg, vitamin B.P, 10,500 mg, choline chloride USP, 125,500 mg, 15,100 mg, iodine B.P, 300 mg, copper B.P, 1,000 mg, zinc USP, 17,555 mg, cobalt B.P, 50 mg, and DL-methionine B.P, 50,500 mg.

#### 2.3.1 Experimental design for probiotic feed trial

Acclimatized fish were allocated randomly into 12 aquaria (60 × 35 × 35 cm) at a density of 10 fish per aquaria for four treatment groups, each having three replicates. All *L. rohita* groups were maintained in properly aerated, fresh, de-chlorinated water at 25 ± 1°C with 25% water exchange daily, and rearing was conducted at a normal day and night (12:12 h) cycle. Water quality was assured by monitoring pH, temperature, dissolved oxygen, and total ammonia concentration daily and found within a range suitable for *L. rohita*. All treated groups of *L. rohita* Tgc, Tbc, and Tmc groups were fed a basal diet added with probiotic *G. candidum* QAUGC01, B*. cereus* QAUBC02, and a mixture of both probiotics, respectively, while the Tc control group was fed with non-supplemented basal diet. All groups were fed at the rate of 3% of their body weight twice a day. Fecal material and undigested food were removed with water to avoid water deterioration on a daily basis.

### 2.4 Fish sampling

The final body weight of every fish was calculated for determination of growth performance at 45 and 90 days. Blood was drained by the tail ablation method and collected in VACUETTE^®^ EDTA K3 (Thomas Scientific, United States) tubes for further analysis by using a hematological analyzer (Sysmex KX21NTM). The hematological parameters studied were red blood cells (RBCs), hemoglobin (HGB), hematocrit (HCT), mean corpuscular hemoglobin (MCH), mean corpuscular hemoglobin concentration (MCHC), MCV (mean corpuscular volume), WBCs (white blood cells), platelets (PLT), and lymphocytes (Lym).

Before sampling, fish were starved for 24 h, and six fish from each aquarium (*n* = 18) were collected randomly, anesthetized with MS-222 (60 mgL^−1^), swabbed with 100% ethanol, and dissected afterward by using a sterile dissection kit in sterile conditions.

#### 2.4.1 Microbial analysis

Whole intestines from the pyloric portion to the rectum were collected in sterile screw-capped collection tubes in three replicates. The contents of the gut were squeezed out and washed with distilled water to remove feed residues; 1 gm of the homogenized intestinal material was used for microbial evaluation. It was carried out for one fish from each replicate.

#### 2.4.2 Enzymatic analysis

Three fish from each group (one from every replicate) were used to determine intestinal enzyme concentrations (amylase, protease, and lipase). The fish intestinal samples were homogenized in phosphate buffer and centrifuged at 15,000 rpm at 4°C for 15 min. The supernatant was removed and kept until analysis at 4°C.

#### 2.4.3 Fish carcass analysis

Proximate analysis was performed by taking six samples from each dietary group, which were further analyzed by AOAC (2000) standard methods. Ash content was determined by placing 2 g of the sample in a muffle furnace and heated at 600°C for 24 h, while crude protein content was determined through the micro-Kjeldahl method after acid digestion of samples. Moreover, total fat content was determined without acid hydrolysis, through the hexane extraction method with the use of a Soxhlet apparatus (Sutharshiny and Sivashanthini, 2011).

The fish carcass was analyzed for biochemical analysis by proximate analysis to observe the nutritional value, and all samples were stored at −20°C for further processing. Growth performance in terms of percent weight gain, SGR, and FCR was measured (Firouzbakhsh et al., 2011).

#### 2.4.4 Preparation of the gut for enzymatic analysis

Protease activity from the gut sample was determined by using 0.65% casein as the substrate, and 1 ml of the sample was incubated with casein at 37°C for 30 min. The filtrate obtained by Whatman filter paper (08 µm) was mixed with 500 mM Na_2_CO_3_ (5 ml) and 0.5 mM Folin and Ciocalteu’s reagent (1 ml). The mixture was incubated for 30 min at 37°C followed by absorbance at 660 nm using a UV–visible spectrophotometer (Shimadzu, Japan). The reaction was ended by using 110 mM trichloroacetic acid (Ibrar et al., 2017). Amylase activity was determined by a method based on 3, 5-dinitrosalicylic acid. Maltose was used as a standard, and reducing sugars are estimated at 560 nm. One amylase unit was defined as the amount of enzyme/mL filtrate that released 1 μg reducing sugar/minute (Bernfeld, 1955; Areekijseree et al., 2004). The activity of cellulase A digestive enzyme was determined using the procedure of Denison and Kohen (1977) with some changes. Citrate phosphate buffer (0.1M) was maintained at pH 5. The reaction mixture comprises 1 ml of carboxymethyl cellulase solution. It was prepared by dissolving 1 g CMC in 100 ml of H_2_O. 1 ml of an appropriate enzyme solution and 1 ml of citrate buffer (0.1 M) were added and incubated for 30 min at 50°C. Afterward, test tubes containing 3 ml DNS were boiled for 15 min to which was mixed 1 ml of sodium potassium tartrate and left to cool. The reducing sugar glucose was measured at 540 nm. One-unit cellulase activity was defined as the amount of enzyme/mL culture filtrate that released 1 mg glucose/minutes.

### 2.5 Determination of intestinal microbiology of fish

Fish intestinal samples were collected at the end of the dietary trial. One gram of the intestinal sample in phosphate buffer with pH 7.4 was homogenized. Centrifugation of the mixture was carried out at 6,000 rpm for 5 min at 4°C, and the supernatant was collected in another sterile falcon tube for advanced analysis.

Gut microbiology was assessed by the culture-dependent method by using five different media, namely, TSA (general purpose), M17 (*Lactococcus*), MRSA (*Lactobacilli*), MacConkey (*Enterobacteriacea*e), and OGA (yeast and fungus). Gut microbiology was carried out by the serial dilution method (David et al., 2014).

#### 2.5.1 DNA extraction, 16S rDNA and ITS library preparation, and sequencing

DNA of the fishes from the whole intestine weighing 0.2 g without gut contents was extracted by using the FavorPrep™ Stool DNA Isolation Mini kit (Favorgen, Taiwan) as per their recommended protocol. Then, DNA was analyzed qualitatively by using gel electrophoresis, and quantity and purity were assessed by using a NanoDrop 1000 spectrophotometer (Thermo Scientific, United States). It was stored at -20°C for further processing.

The 16S rDNA fragments of the V4 region of bacteria and ITS for the fungus variable region of each sample were generated by PCR using 515F (GTGCCAGCMGCCGCGGTAA)/806R (GGACTACHVGGGTWTCTAAT) and ITS primers (Caporaso et al., 2011). PCR was operated in three replicates by using the Hot Star Taq Plus Master Mix Kit (Qiagen, United States) with a 20 ul reaction mixture. The following conditions of PCR were applied: initial denaturation at 94°C for 3 min, followed by 30 cycles of denaturation at 94°C for 30 s, annealing at 53°C for 40 s, and extension at 72°C for 1 min. Amplification success and relative intensity of bands were determined by using 2% agarose gel. After that, equal proportions of all samples were pooled together and purified by using calibrated AMPure XP DNA beads (Beckman Coulter Genomics GmbH, Bernried, Germany). Then, the pooled and purified PCR product was used to prepare the Illumina DNA library. Sequencing was performed at MR DNA (www.mrdnalab.com, Shallowater, TX, United States) by using an Illumina Miseq platform (Illumina, San Diego, United States) by using a version 3 Miseq reagent kit.

#### 2.5.2 Analysis of 16S rDNA and the ITS dataset

Sequenced raw data were processed bioinformatically by using the MR DNA pipeline. In summary, raw forward and reverse sequences were joined by using the Fastq join algorithm, considering a maximum of 50 nucleotides overlapped and not more than one mismatch to develop paired-end reads. After alignment, paired-end sequences were depleted of barcodes and primers, then sequences <150bp were removed, and sequences with ambiguous base calls were removed. After that, the sequences were denoised, and individual sample paired-end sequence files were merged into a single FASTA file. OTUs were generated with 97% homology, and chimeras were removed by using reference-based and *de novo* approaches. Operational taxonomic units (OTUs) were defined by clustering at different similarity cut-off levels. Final OTUs were taxonomically classified using BLAST against a curated database derived from RDPII, RDPI, and RDPI (DeSantis et al., 2006) and NCBI (www.ncbi.nlm.nih.gov , http://rdp.cme.msu.edu). The 16S rDNA sequenced data in this project were deposited in the Sequence Read Archived database in the NCBI under accession number PRJNA95448.

### 2.6 Challenge test against *Staphylococcus aureus*

*Staphylococcus aureus* is one of the fish intestinal pathogens that causes foodborne infection, and it is multidrug-resistant as well. The control and experimental groups were treated with *S. aureus* to investigate the anti-pathogenic effect of probiotics. At the end of a 90-day feeding experimental trial, two groups Tc (control) and Tmc (*B. cereus* and *G. candidum*) were challenged by *S. aureus* (100 µL with 10^8^ CFU/ml), and each treatment was carried out in duplicate, with six fishes in each aquarium. The basal group on the control diet was assigned as a positive control and also underwent a challenge test. *L. rohita* survival rate was observed for a week for any disease symptoms and mortality.

Survival rate % = Number of fish at the end of experiment/number of fish at the beginning of experiment × 100.

### 2.7 Statistical analysis

The data were investigated by one-way analysis of variance (ANOVA) using XLSTAT. Differences between the means were tested by Tukey’s multiple ranges and Duncan’s tests. The relationship between biochemical and physiological parameters was analyzed by Pearson’s correlation using XLSTAT. Alpha diversity was applied for analyzing the complexity of species diversity among samples using OTUs, Shannon, and Simpson indices. Venn diagrams were generated by using the Venn diagram package in R version 3.4.3 (http://www.r-project.rog). The hierarchically clustered heat map figures were generated by using HemI (Heatmap Illustrator, version 1.0). Differences were represented as significant when *p* < 0.05. The results were presented as means ± SD (standard deviation).

## 3 Results

### 3.1 Fish growth and feed utilization efficiency

The different probiotic supplements have shown a significant impact on percent body weight gain. The highest percent body weight was observed for mixed culture (Tmc) after a 90-day feeding trial. The highest specific growth rate was observed for mixed culture (Tmc) which was significantly higher (*p* < 0.05) than that of the control after the 45- and 90-day trials. FCR with significant positive variation was observed for mixed culture (Tmc) compared to control after 45 days, while at the 90 day, significant variation (*p* < 0.05) was observed for Tbc, and the remaining treatments showed non-significant variation compared to that of the control (Table 2).

**TABLE 2:** Impact of feeding probiotic microorganisms (10^9^ CFU/gm diet) in single and mixed culture on growth performance of *Labeo rohita.*

Parameter	Days	Control (Tc)	*G. candidum* (Tgc)	*B. cereus* (Tbc)	Mixed culture (Tmc)
Growth %	45	47.31 ± 3.09^c^	68.68 ± 1.16^b^	70.29 ± 2.9^b^	123.29 ± 2.79^a^
	90	85.27 ± 3.11^c^	108.0 ± 2.47^b^	83.62 ± 3.26^c^	141.48 ± 1.80^a^
SGR	45	1.28 ± 0.07^c^	1.74 ± 0.02^bc^	1.74 ± 0.05^ab^	2.67 ± 0.04^a^
	90	0.76 ± 0.11^c^	0.97 ± 0.13^b^	0.78 ± 0.02^bc^	1.09 ± 0.10^a^
FCR	45	3.40 ± 0.04^a^	2.37 ± 0.19^b^	3.46 ± 0.40^a^	2.28 ± 0.07^b^
	90	3.121 ± 0.12^b^	2.33 ± 0.11^b^	3.15 ± 0.25^a^	2.21 ± 0.10^b^

Note: T0/Tc (basal diet), T1/Tgc (QAUGC01), T4/Tbc (QAUBC01), and T7 (QAUGC01 + QAUBC01). These tabular data are represented as Mean ± SD (*n* = 18). The alphabets a, b, c above the values show the significance difference between treatments calculated by one way ANOVA (p < 0.05), followed by Tukey’s and Duncan’s analyses (a>b>c).

% weight gain = final body weight (wf) − initial body weight (wi)/initial body weight × 100.

Specific growth rate = *ln* of final body weight (*ln* wf) − *ln* of initial body weight (*ln* wi)/duration of experiment days × 100.

FCR = net consumed feed (g)/net weight gain.

FCE % = 1/FCR×100.

### 3.2 Fish carcass composition

The highest accumulated body protein at 45 days was observed in groups Tgc and Tmc, which was significantly higher (*p* < 0.05) than that of the control, while accumulated body fats in Tgc and Tbc groups were significantly higher (*p* < 0.05) than those in Tc and Tmc groups after 45 days. After 90 days, all treated groups showed a significantly higher percentage of protein and fat than the control group, and Tbc showed the highest value. Minimum protein content after 90 days was observed in Tgc, and minimum fat was found in Tc after 90 days. Carcass ash content showed non-significant differences after 45 days and after 90 days except for Tbc, which showed significantly lower ash content than Tc, Tgc, and Tmc after 90 days (Table 3).

**TABLE 3:** Impact of feeding probiotic microorganisms (10^9^ CFU/gm diet) in single and mixed culture on intestinal enzyme activity and body dry mass chemical composition of *Labeo rohita.*

Group		Control (Tc)	*G. candidum* (Tgc)	*B. cereus* (Tbc)	Mixed culture (Tmc)
Parameter	Days				
Protein %	45	65.5 ± 0.29^b^	74.38 ± 0.05^a^	66.5 ± 1.1^b^	74.38 ± 0.23^a^
	90	70.00 ± 1^c^	68.38 ± 0.46^d^	83.10 ± 0.5^a^	78.75 ± 0.80^b^
Fats %	45	9.00 ± 0.17^b^	12.3 ± 0.12^a^	12.30 ± 1.1^a^	8.60 ± 0.29^b^
	90	18.3 ± 1.1^d^	28.6 ± 1.1^a^	28.60 ± 1.1^b^	21.3 ± 0.9^c^
Ash %	45	14 ± 0.24^a^	13.5 ± 0.12^a^	12 ± 1.0^a^	12 ± 0.33^a^
	90	16 ± 1.4^a^	15.5 ± 1.5^ab^	13.2 ± 1.4^b^	14 ± 0.5^ab^
Protease (specific activity U mg^−1^)	45	0.186 ± 0^b^	0.187 ± 0^b^	0.185 ± 0^b^	0.199 ± 0^a^
	90	0.16 ± 0^c^	0.13 ± 0^d^	0.177 ± 0^b^	0.22 ± 0^a^
Amylase (specific activity U mg^−1^)	45	0.31 ± 0^b^	0.22 ± 0^c^	0.39 ± 0^a^	0.22 ± 0^c^
	90	0.32 ± 0^b^	0.25 ± 0^c^	0.413 ± 0^a^	0.24 ± 0^d^
Cellulase (specific activity U mg^−1^)	45	0.154 ± 0^b^	0.098 ± 0^c^	0.213 ± 0^a^	0.120 ± 0^bc^
	90	0.163 ± 0^bc^	0.139 ± 0.01^d^	0.265 ± 0^a^	0.152 ± 0.01^c^

Note: T0/Tc (basal diet), T1/Tgc (QAUGC01), T4/Tbc (QAUBC01), and T7 (QAUGC01 + QAUBC01). This tabular data are represented as Mean ± SD (*n* = 18). The alphabets a, b, c, d above the values show the significance difference between treatments calculated by one way ANOVA (p < 0.05), followed by Tukey’s and Duncan’s analyses (a>b>c>d).

### 3.3 Digestive enzyme activity

Tmc (mixed culture) showed significantly high protease activity (*p* < 0.05) throughout the feeding trial. Amylase and cellulase activity of the Tbc group was significantly higher than that of the control and the rest of the two treated diets (Tgc and Tmc) throughout the 90-day trial (Table 3).

### 3.4 Hematological parameters

Maximum RBCs, Hgb, MCH, MCHC, platelets, WBCs, and lymphocyte count after 45 days were observed in Tmc which was significantly (*p* < 0.05) higher than those in the control and other treatments (Tgc and Tbc), except for HCT and MCV counts, which were maximum in Tbc treatment. After 90 days, the highest count of RBCs, WBCs, Hgb, HCT, MCH, MCV, and lymphocytes was recorded for Tm except for MCHC and platelets, which were maximum in the Tgc group after 90 days, while no significant difference in MCH level as compared to that of the control was observed in the fish group fed with Tgc (Table 4).

**TABLE 4:** Impact of feeding probiotic microorganisms (10^9^ CFU/gm diet) in single and mixed culture form on hematological parameters of *Labeo rohita.*

Parameter	Day	Control (Tc)	*G. candidum* (Tgc)	*B. cereus* (*Tbc*)	Mixed culture (Tmc)
RBCs (10^6^μL^−1^)	45	1.83 ± 0.01^c^	2.21 ± 0.06^b^	2.25 ± 0.14^b^	2.43 ± 0.02^a^
	90	1.06 ± 0.01^d^	1.96 ± 0.03^c^	2.20 ± 0.15^b^	2.75 ± 0.01^a^
Hgb (g/dl)	45	6.7 ± 0.58^b^	7.7 ± 0.29^b^	7.4 ± 0.8^b^	9.7 ± 0.35^a^
	90	4.6 ± 0^.^6^d^	7.9 ± 0.1^c^	8.7 ± 0.1^b^	11.5 ± 0.5^a^
HCT (%)	45	24.0 ± 0.46^bc^	23.7 ± 0.23^c^	31.5 ± 0.4^a^	25.3 ± 0.58^b^
	90	13.26 ± 1.2^d^	19.90 ± 1^c^	27.4 ± 0.7^b^	39.13 ± 1.2^a^
MCH (pg)	45	36.6 ± 0.58^b^	37.5 ± 0.17^b^	32.9 ± 0.45^c^	39.7 ± 9.67^a^
	90	42.66 ± 1.8^a^	41.50 ± 0.7^a^	38.6 ± 0.8^b^	41.23 ± 1.0^a^
MCHC (g dl^−1^)	45	27.9 ± 0.52^c^	33.4 ± 0.17^b^	23.5 ± 0.9^d^	39.9 ± 0.29^a^
	90	34.20 ± 1.5^b^	38.80 ± 0.9^a^	31.1 ± 1.7^c^	30.70 ± 1.5^c^
MCV (10^–15^ L)	45	131.1 ± 0.5^b^	114.2 ± 0.6^c^	139.6 ± 1.5^a^	100.0 ± 0.3^d^
	90	123.3 ± 0.8^b^	102.8 ± 1.1^c^	125.4 ± 1.1^b^	143.54 ± 1.1^a^
Plt (10^3^/µL)	45	13.0 ± 1^d^	19.0 ± 1^c^	22.0 ± 1^b^	54.0 ± 1^a^
	90	86.6 **±** 1.5^b^	212 **±** 2.0^a^	65.8 ± 1.6^c^	60.3 ± 1.8^d^
WBCs (10^3^/µL)	45	178.4 ± 0.58^d^	184.2 ± 0.35^c^	193.6 ± 1.1^b^	223.8 ± 0.58^a^
	90	142.2 ± 2.1^c^	240.0 ± 1.0^b^	239 ± 1.9^b^	253.6 ± 2.0^a^
Lym (%)	45	97.6 ± 0.1^a^	97.7 ± 0.5^a^	98.9 ± 1^a^	97.9 ± 1^a^
	90	77.66 **±** 0.9^b^	97.30 **±** 0.8^a^	97.8 ± 1.6^a^	95.10 ± 4.9^a^

Note: T0/Tc (basal diet), T1/Tgc (QAUGC01), T4/Tbc (QAUBC01), and T7 (QAUGC01 + QAUBC01). This tabular data are represented as Mean ± SD (*n* = 18). The alphabets a, b, c, d above the values show the significance difference between treatments calculated by one way ANOVA (*p* < 0.05), followed by Tukey’s and Duncan’s analyses (a>b>c>d).

### 3.5 Relationship between biochemical and physiological variables

All blood parameters and indices showed a positive correlation with growth rate or body weight gain except MCV; of them WBCs, RBCs, Hgb, and HCT showed a significant positive correlation (*p* < 0.05). SGR and FCE also showed a significant (*p* < 0.05) positive correlation, while FCR showed a significant negative correlation with growth rate. Protease activity showed a positive correlation with growth parameters, except for FCE and protein percentage in the carcass. Amylase showed a significantly negative correlation with growth parameters, except for FCR to which it showed a significant positive correlation. Cellulase showed a significant positive correlation with carcass protein, fats, and FCR. There is also a positive correlation between protein and amylase activity (Supplementary Tables S4A, B).

### 3.6 Fish gut microbial diversity by culturing method

The presumptive total aerobic count of the Tmc and Tbc (1.01× 10^8^ & 7.23× 10^7^) groups fed with mixed culture (containing two probiotic strains) and *B. cereus* was higher than that of the Tgc (*G. candidum*) (3.43× 10^7^) group and was lower than that in control (5.93× 10^7^). Presumptive LAB count was increased by *G. candidum* in a single form (9.92× 10^7^), while it was lower in Tmc and Tbc than in the control. Probiotic feeding reduces *Enterobacteriaceae* (coliforms) compared to that of control. The assumed yeast count was higher in treated groups, but Tgc showed the highest count (8.41× 10^7^) of yeast than control fed with basal diet. The assumed *Enterococcus* count was higher in Tc (2.87× 10^8^) than in treated groups which were 5.82× 10^7^, 6.09× 10^7^, and 9.59× 10^7^ (Table 5).

**TABLE 5:** Cumulative table showing colony forming units per gram on culturing media.

Treatment	Tryptic soy (TSA)	MacConkey (MC)	M17	MRS	Oxytetracycline (OGA)
Tc	5.93E+07	1.64E+07	2.87E+08	4.64E+06	364E+05
Tgc	3.43E+07	2.25E+06	5.82E+07	9.92E+07	8.41E+07
Tbc	7.23E+07	2.14E+06	6.09E+07	4.45E+06	6.91E+07
Tmc	1.01E+08	5.05E+06	9.59E+07	1.68E+06	3.77E+06

*TC (basal diet), Tgc (*G. candidum* QAUGC01), Tbc (*B. cereus* QAUBC02), and T7 (*G. candidum* QAUGC01 co-culture with *B. cereus* QAUBC02).

### 3.7 Fish gut microbial diversity

After filtering the low-quality reads, the barcodes, primers, chimeras, and longer homopolymers were removed, omitting all chloroplast eukaryotes and cyanobacteria sequences and normalizing the data. In total, 46,598–71,409 bacterial sequences and 14,110–466,192 fungal sequences were collected from four samples. The studied four samples resulted from 254,847 bacterial and 879,304 fungal sequences collectively. The average number of bacterial sequences from all samples at various similarity levels was 217,978 ± 11,060 (99–100% homology), 14,350 ± 3,661 (98% homology), 18,294 ± 1,584 (97% homology), 2,485 ± 225 (96% homology), and 437 ± 55 (95% homology), while the average number of fungal sequences from all samples at various similarity levels was 822,084 ± 237,811 (99–100% homology), 28,989 ± 3,683 (98% homology), 15,441 ± 1,323 (97% homology), 583 ± 152 (96% homology), and 1,426 ± 245 (95% homology).

After delineating the resulting sequence homology, a total of 594 bacterial and 245 fungal OTUs were developed with different levels. Each sample contained 146–177 bacterial and 95–169 fungal OTUs. The richness of groups Tgc fed with *G. candidum* and Tmc fed with a mixed culture of *G. candidum* and *B. cereus* was lower than that of the Tbc fed with *B. cereus* and Tc fed with a basal diet. The microbial community was more diverse in the gut of the Tgc and Tmc groups than in the other two groups, Tc and Tbc, as calculated using Shannon indices. Bacterial species in the Tgc and Tmc groups were evenly distributed as compared to the Tc and Tbc groups, while fungal species in Tc, Tgc, and Tmc were evenly distributed as compared to Tbc as evaluated by using Simpson’s indices (Table 6).

**TABLE 6:** Alpha diversity profile of control and probiotic-treated samples.

Sample ID	Number of read		Number of OTUs		Shannon index		Simpson index		Observed species	
	Bacteria	Fungi	Bacteria	Fungi	Bacteria	Fungi	Bacteria	Fungi	Bacteria	Fungi
Tc	67926	381936	158	169	0.70	0.57	0.23	0.78	158	169
Tgc	71409	14125	150	95	1.33	1.44	0.64	0.71	150	95
Tbc	68914	468409	177	160	0.67	0.20	0.24	0.05	177	160
Tmc	46598	17143	146	96	1.44	1.44	0.67	0.71	146	96

Number of analyzed reads, diversity richness (OTUs), diversity index (Shannon and Simpson) for 16S rDNA, and ITS sequencing libraries of probiotic-treated and control samples.

### 3.8 Impact of probiotics on intestinal bacterial community

**FIGURE 1:**
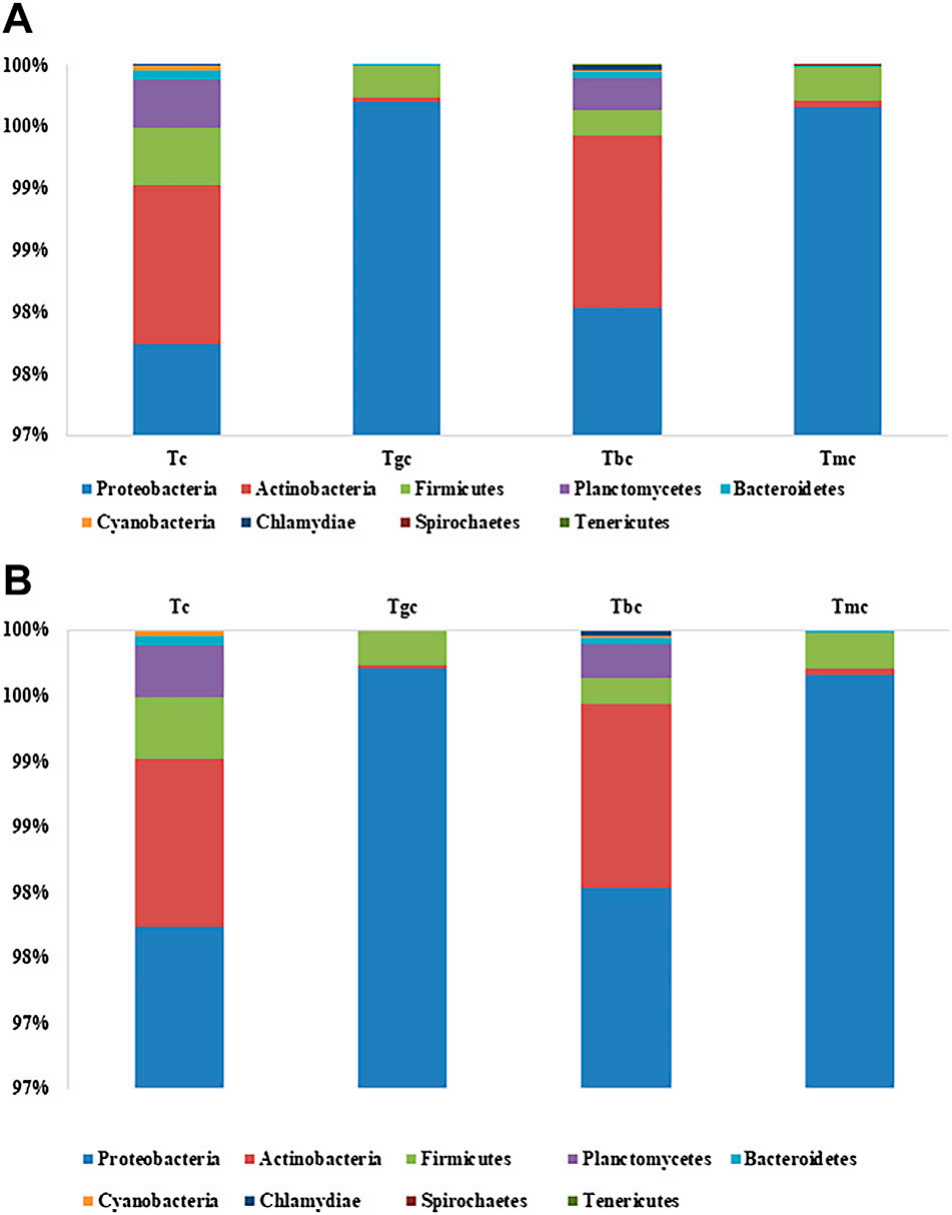
**(A)** Relative abundance (%) gut bacterial diversity at the phylum level after probiotic feeding. Tc: treatment control, gc: *G. candidum*, Bc: *Bacillus cereus*, mc: mix culture. **(B)** Relative abundance (%) of gut bacterial diversity at the species level after probiotic feeding, and species with a percentage less than 0.1% were excluded.

The bacterial community composition dominance trend at the phylum level was nearly the same both in the probiotic-treated and -controlled groups. The OTUs were classified at different phylogenetic levels such as phylum, class, order, genus, and species. At the phylum level, the OTUs were classified into 10 bacterial phyla ranging from four to nine in each sample (Figure 1A). Within these phyla, eight to 18 bacterial classes were recorded. The number of OTUs classified at the genus level ranged from 46 to 84, while collectively 111 genera were recorded. The six phyla present in the control group were *Proteobacteria* (97.74%), *Actinobacteria* (1.28%), *Firmicutes* (0.46%), *Planctomycetes* (0.39%), *Bacteroidetes* (0.07%), and *Chlamydia* (0.001%). The Tbc group showed a maximum of nine phyla, and they are *Proteobacteria* (97.98%), *Actinobacteria* (1.39%), *Firmicutes* (0.20%), *Planctomycetes* (0.25%), *Bacteroidetes* (0.05%), *Chlamydia* (0.04%), *Verrucomicrobia* (0.05%), *Spirochaetes* (0.001%), and *Tenericutes* (0.001%). Out of them, *Verrucomicrobia*, *Spirochaetes*, and *Tenericutes* were just present in the Tbc group. The Tgc group represented four phyla, namely, *Proteobacteria* (99.70%), *Firmicutes* (0.25%), *Actinobacteria* (0.03%), and *Bacteroidetes* (0.005%). The group Tmc fed with mixed probiotics represents five phyla, namely, *Proteobacteria* (99.66%), *Firmicutes* (0.27%), *Actinobacteria* (0.04%), *Bacteroidetes* (0.008%), and *Spirochetes* (0.004%). *Proteobacteria* represented the first dominant OTU in all groups, while in Tc and Tbc, γ class, and in Tgc and Tmc, β class of *Proteobacteria* were dominant.

In phylogenetic classification at the species level at 3% divergence (97% similarity), OTUs were classified into 63–103 species in each sample, totaling 141 species collectively. Tc and Tbc showed a higher number of species than Tgc and Tmc, and there were a total of 103 species in Tbc, while 94 species in Tc. After omitting species with relative abundance (<0.1), 14 species remained in Tbc and 19 in Tc. The dominant species were *Pseudomonas psychrophila* in Tc (87.21%) and Tbc (86.53%), *Pseudomonas plecoglossicida* in Tc (0.82%) and Tbc (0.40%), *Pseudomonas spp.* In Tc (0.51%) and Tbc (0.48%), *Achromobacter xylosoxidans* in Tc (0.32%) and Tbc (0.28%), *Pseudomonas trivialis* in Tc (0.44%) and Tbc (0.46%) *Pseudomonas syringae* in Tc (2.18%) and Tbc (3.12%), *Pseudomonas fragi* in Tc (3.87%) and Tbc (5.64%), *Acidothermus cellulolyticus* in Tc (0.95%) and Tbc (1.22%), *Rhodobacter spp.* In Tc (0.52%) and Tbc (0.29%), *Mycobacterium sp.* in Tc (0.12%), *Clostridium spp.* in Tc (0.14%), *Rhodopseudomonas palustris* in Tc (0.21%), *Singulisphaera spp.* in Tc (0.16%), *Bosea thiooxidans* in Tc (0.27%), and *Staphylococcus saprophyticus* in Tc (0.15%). The genus *Pseudomonas* was dominantly recovered from both Tc and Tbc groups, indicating that feeding of *B. cereus* in a single form did not alter the gut microbiological profile.

Tgc and Tmc showed reduced abundance at the species level than Tc and Tbc, and there were a total of 63 species in Tgc and 64 species in Tmc. After omitting species with relative abundance (<0.1), 13 species remained in Tgc and 14 in Tmc. Dominant species were *Achromobacter xylosoxidans* in Tmc (48.89%) and Tgc (51.82%), *Klebsiella oxytoca* in Tmc (25.53%) and Tgc (25.88%), *Serratia quinivorans* in Tmc (12.53%) and Tgc (13.06%), *Raoultella ornithinolytica* in Tmc (4.34%) and Tgc (4.38%), *Pseudomonas trivialis* in Tmc (3.82%) and Tgc (0.17%), *Achromobacter spp.* in Tmc (2.82%) and Tgc (2.70%), *Stenotrophomonas spp.* in Tmc (0.22%) and Tgc (0.44%), *Pseudomonas psychrophila* in Tmc (0.29%) and Tgc (0.23%), *Enterobacter spp.* in Tmc (0.23%) and Tgc (0.22%), *Bacillus szutsauensis* in Tmc (0.17%), and *Paenibacillus lactis* in Tmc (0.12%) and Tgc (0.10%). The dominant genus in Tmc and Tgc was *Achromobacter*, and the overall profile of the gut was altered by feeding *G. candidum* and a mixture of *G. candidum* and *B. cereus* (Figure 1B).

### 3.9 Impact of probiotics on intestinal fungal community

**FIGURE 2:**
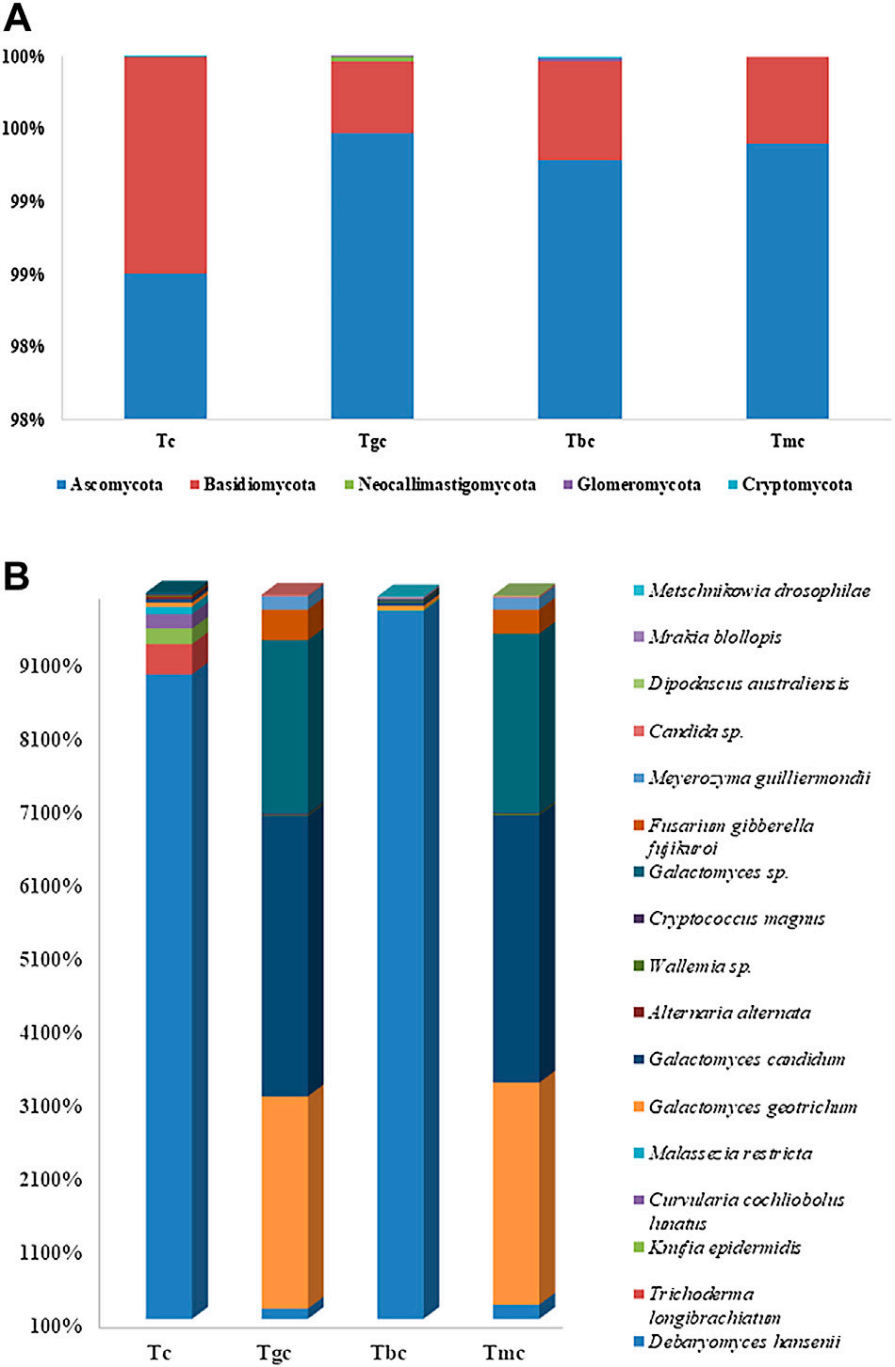
**(A)** Relative abundance (%) gut fungal diversity at the phylum level after probiotic feeding. **(B)** Relative abundance (%) of gut fungal diversity at the species level after probiotic feeding, and species with a percentage less than 0.1% were excluded.

The fungal community composition dominance trend at the phylum level was nearly the same both in the probiotic-treated and -controlled groups. The OTUs were classified at different phylogenetic levels such as phylum, class, order, genus, and species. At the phylum level, the OTUs were classified into five fungal phyla, ranging from two to five in each sample (Figure 2A), and within these phyla, 15 bacterial classes were recorded. The number of OTUs classified at the genus level ranged from 28 to 41, while collectively 44 genera were recorded. The control group showed five phyla, namely, *Ascomycota* (98.50%), *Basidiomycota* (1.48%), *Neocallimastigomycota* (0.002%), *Glomeromycota* (0.001%), and *Cryptomycota* (0.0005%). Tbc group represented five phyla, namely, *Ascomycota* (99.28%), *Basidiomycota* (0.68%), *Neocallimastigomycota* (0.002%), *Glomeromycota* (0.01%), and *Cryptomycota* (0.01%). The Tgc group represented four phyla, namely, *Ascomycota* (99.46%), *Basidiomycota* (0.49%), *Neocallimastigomycota* (0.02%), and *Glomeromycota* (0.007%). The Tmc group showed just two phyla, *Ascomycota* (98.40%) and *Basidiomycota* (0.59%). In both probiotic-treated and control groups fed with basal diet, *Ascomycota* phylum class *Saccharomyces*, was dominant.

In phylogenetic classification at the species level at 3% divergence (97% similarity), OTUs were classified into 35–57 species in each sample while 64 species were classified collectively. Tc and Tbc showed a higher number of species than Tgc and Tmc, and there were a total of 51 species in Tbc, while 57 species in Tc. After omitting species with relative abundance (<0.1), 10 species remained in Tbc and 11 in Tc. The control group (Tc) and Tbc were abundant in *Debaryomyces hansenii* in Tc (88.61%) and Tbc (97.22), followed by *Knufia epidermidis* in Tc (2.09%) and Tbc (0.16%), *Galactomyces candidum* in Tc (0.50%) and Tbc (0.41%), *Galactomyces geotrichum* in Tc (0.55%) and Tbc (0.54), *Wallemia spp.* in Tc (0.19%) and Tbc (0.16%), *Cryptococcus magnus* in Tc (0.18%) and Tbc (0.16), *Trichoderma longibrachiatum* in Tc (4.11%), *Curvularia cochliobolus lunatus* in Tc (1.95%), and *Malassezia restricta* in Tc (1.00%). The genus *Debaryomyces* dominantly recovered from both Tc and Tbc, which indicates that feeding of *B. cereus* in a single form did not alter the gut microbiological profile.

Tgc and Tmc showed reduced abundance at the species level same as in the bacterial community than Tc and Tbc, and there were a total of 35 species both in Tgc and Tmc. After omitting species with relative abundance (<0.1), nine species remained in Tgc and 10 in Tmc. Tgc and Tmc groups were abundant in *Galactomyces candidum* in Tmc (36.32%) and Tgc (38.09%), followed by *Galactomyces geotrichum* in Tmc (30.18%) and Tgc (28.85%), G*alactomyces spp.* in Tmc (24.32%) and Tgc (23.55%), *Fusarium gibberella fujikuroi* in Tmc (3.28%) and Tgc (4.155), *Debaryomyces hansenii* in Tmc (2.95%) and Tgc (2.40%), *Meyerozyma guilliermondii* in Tmc (1.60%) and Tgc (1.80%), *Wallemia spp.* in Tmc (0.21%) and Tgc (0.14%), and *candida spp.* in Tmc (0.21%) and Tgc (0.20%) (Figure 2B). The dominant genus in Tmc and Tgc was *Galactomyces*, and the overall yeast and fungal profiles of the gut were altered by feeding *G. candidum* and a mixture of *G. candidum* and *B. cereus* (Figure 2B)*.*

### 3.10 Shared and unique microbial populations

**FIGURE 3:**
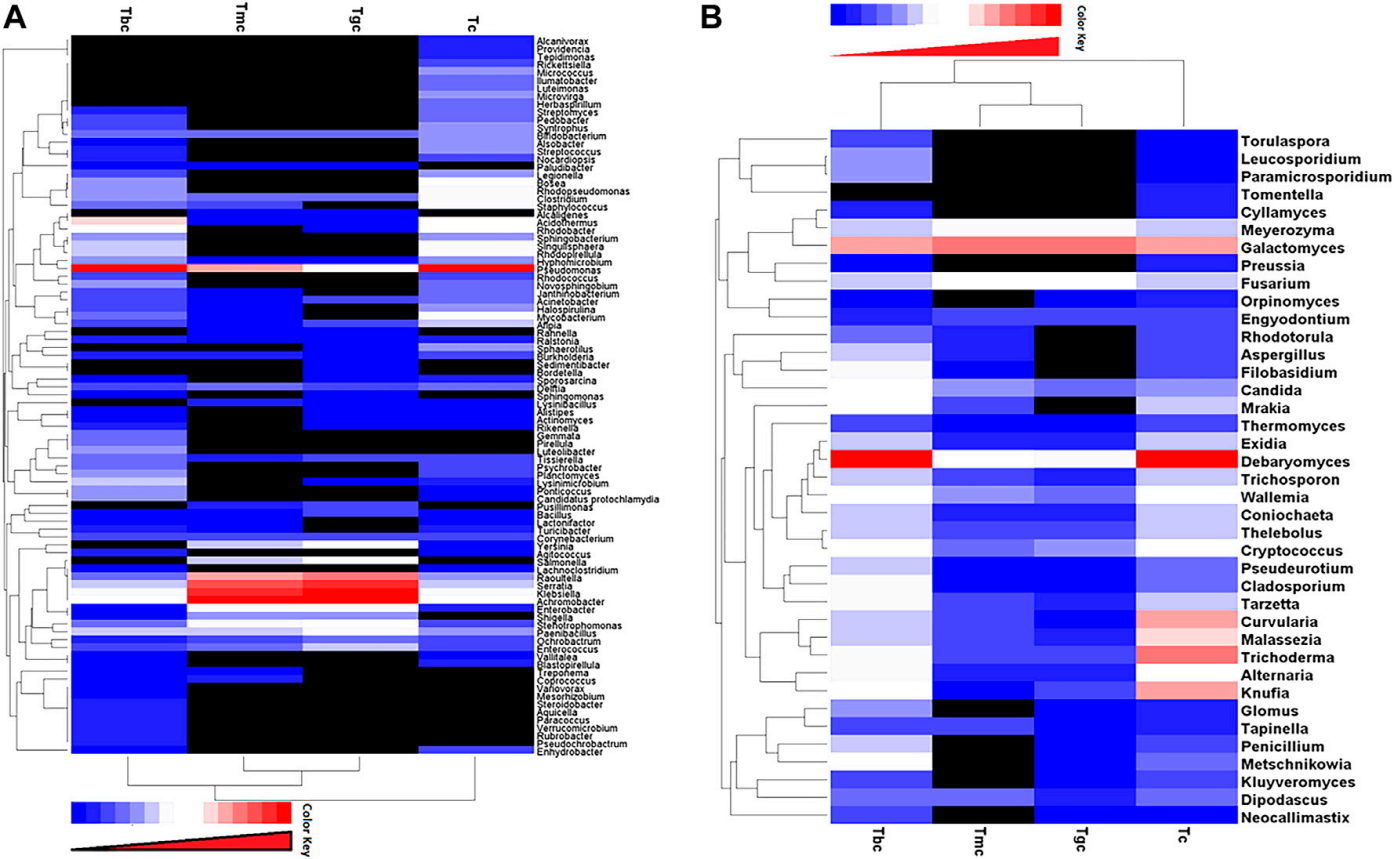
**(A**,**B)** Distribution of bacterial and fungal genera among treated and control groups. Double hierarchically clustered heatmap showing the bacterial and fungal distribution among the composition. The heatmap depicts the OTU counts of each bacterial and fungal genus (clustering on the *Y*-axis) within each sample (clustering on *X*-axis). Relative values for bacterial and fungal genera are depicted by the color intensity with the color key shown in the figure. **(A)** Bacterial community; **(B)** fungal community.

The hierarchically clustered heatmap analysis based on the bacterial community profiles at the genus level depicted that the bacterial communities in the GIT of Tmc and Tgc fed with *G. candidum* both in single and combination form with *B. cereus* shared higher similarity than those in Tbc and Tc. So, Tmc and Tgc clustered first together, then they clustered with Tbc, and after that, they clustered with Tc. Tc is more distant or dissimilar from the Tgc- and Tmc-treated groups than Tbc (Figure 3A). The hierarchically clustered heatmap analysis based on the fungal community profiles at the genus level depicted almost the same pattern as in the case of the bacterial community. Fungal communities in the GIT of Tmc and Tgc shared higher similarities than those of Tbc and Tc. So, Tmc and Tgc clustered first together, then they clustered with each Tbc, and then with Tc. Tc is more distant or dissimilar from the Tgc- and Tmc-treated groups than Tbc, and the distance between Tc and treated groups in the case of fungal communities is higher than that between the bacterial communities (Figure 3B).

**FIGURE 4:**
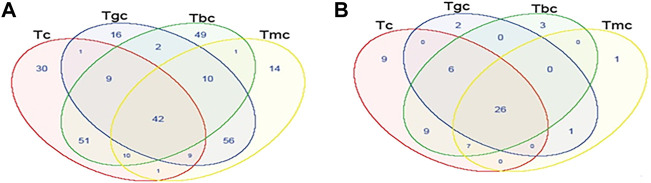
Shared OTU analysis of the treated and control groups. Venn diagram showing the unique and shared OTUs (3% distance level) in the probiotic-treated and control groups. **(A)** Venn diagram represents the shared and unique bacterial community among groups. **(B)** Venn diagram represents the shared and unique fungal community among groups.

Shared and unique OTUs between treated and control groups were analyzed by generating Venn diagrams. The Venn diagram for the bacterial community showed that OTUs for 42 species (13%) were shared in all four groups. Highest shared OTUs for 56 species (18%) were present in the Tgc and Tmc groups, while the Tbc and Tc groups showed (16.9%) a shared community. The unique bacterial community present in Tbc (16%) was the highest followed by Tc (9.9%) (Figure 4A). The fungal community when analyzed by the Venn diagram showed that there are shared OTUs for 26 species (40%) in all four groups. Tbc and Tc groups shared a 14% fungal community, while Tc's unique community was also 14%. Although all groups showed a higher level of shared community, the richness of specific fungal species was quite different among the groups (Figure 4B).

### 3.11 Resistance against *S. aureus*

Both the positive control group fed on a basal diet and the treated group fed on (*G. candidum and B. cereus*) were injected with *S. aureus* injection. Fish death was observed in the control group fed on the basal diet, while the treated groups (*G. candidum and B. cereus*) survived at the end of the challenge test. The fishes in the positive control group died because of the infection caused by the pathogen, while the survival of the treated group might be due to the production of antimicrobials, competitive exclusion of the pathogen, or modification of an environmental niche continuing for 7 days.

## Discussion

Feeding practices are very instrumental for raising healthy fishes as it is linked with their survival, productivity, growth trends, and resistance to diseases. It is an accepted fact that probiotic ingestion has profound beneficial effects on aquaculture, but its effect varies depending on the size and age of the fish. Herein, the synergistic effect of locally isolated strains was observed: G. *candidum* isolated from fermented milk and *Bacillus cereus* from the gut of rohu fingerlings were added to the feed to observe the impact on growth, physiology, and microbial diversity of *L. rohita*. Recently, this combination of strains has imparted a positive impact on fry of *L. rohita* in an 11-week trial in terms of improved growth, muscle composition, survival, and protection against diseases (Amir et al., 2018). A limited literature is available on the synergistic effect of yeast and bacterial strains in aquaculture, and the microbial gut modulation is yet to be explored further. Previously conducted studies on different fish species confirmed the beneficial effects of probiotics, and it is suggested that it could be one of the possible solutions for high death rates and insufficient defense during the earlier life stages of fish, such as giant freshwater prawn (Nayak, 2010; Sun et al., 2010; Gupta and Dhawan, 2012) and common carp (Gupta et al., 2014; Tarnecki et al., 2019; Hoseinifar et al., 2018). In the present study, it was found that the mixed culture of *G. candidum* and *B. cereus* showed promising results in both *in vitro* and *in vivo* conditions in terms of growth rate, FCR, and resistance to diseases. It might be due to the mutualism of these two strains which could strongly bind with intestinal mucosa, thus blocking pathogen adhesion and invasion (Mohapatra et al., 2012). The enhanced growth observed during our study might be due to increased production of digestive enzymes, vitamins, reduced stress, nutrient availability, and reduction in pathogens. Similar findings have been reported earlier (Swain et al., 1996; Gomez and Balcazar, 2008; Bolasina et al., 2006). It may be deduced that growth is not only a strain-specific phenomenon, but also it depends on multiple variables, such as digestive system capacity, water quality, and rearing practices (De Silva and Anderson, 1994).

Maximum protein and fat contents were observed in Tgc (*G. candidum*) and Tmc (*G. candidum and B. cereus*). This increment in these contents might be linked to an increase in their formation and deposition due to efficient assimilation of ingested food (Mehrabi et al., 2012; Noveirian and Nasrollahzadeh, 2012). Earlier, Ibrar et al. (2017) found an increase in body fat and protein content when *L. rohita* specimens were reared on *G. candidum-*supplemented diet. The other factors reported to affect body composition are rearing conditions, species of fish, growth conditions, stock density, and level of protein in the diet (Abdel-Tawwab, 2012; Khalil et al., 2012). Ash content was lower in treated groups, but the differences were non-significant. These results are in agreement with reports of a non-significant variation in ash content when Biogen^®^, a commercial probiotic in *Nile tilapia*, was used as a probiotic (Eid and Mohamed, 2008).

Tmc (the co-culture of *B. cereus* and *G. candidum*) positively influenced the hematology of fishes in terms of WBCs, RBCs, Hgb, platelets, MCH, and HCT, while HCT and MCV were maximum in Tbc treatment at 45 days. Improved hematology in fishes fed on probiotic feed is reported by many previous studies (Marzouk et al., 2008; Dahiya et al., 2012; Renuka et al., 2014). The hematocrit value for most of the treatments used in the present study lies within the normal range. Maximum MCHC and platelet count after 90 days were observed in Tgc treatment, which was significantly higher than those of the control. Our data showed that most of the treatments fall in the normal range of MCHC (23–39 gdL^−1^) and MCV was also in the recommended range (100–143 fL/cell), but values were found to be out of range, and these slight variations could be suggested due to various reasons (George-Gay and Parker, 2003).

The intestinal enzymes play a significant role in the digestion of feed. Maximum protease activity which was significantly higher than that of the control was observed in Tmc (*B. cereus* and *G. candidum*). This might be due to the interaction of the co-culture and modulated intestinal resident microbial community which enhanced protease expression. Earlier, Mohapatra et al., (2012) reported that a combination of bacterial and yeast strains as probiotics immensely enhanced protease activity. Low enzyme activity in the initial life stages due to an immature digestive system can be improved by using probiotics having immense enzymatic potential. Amylase and cellulase activities were found to be significantly higher than those in control. The same results were observed for the activity of the Tbc (*B. cereus*) group at 45 and 90 days, which may be linked to the composition of feed that is mainly composed of carbohydrates. It is also possible that the intestine is endogenously loaded with these enzyme-producing bacteria, or it may be because *Bacillus* sp. is a putative probiotic known for its enzymatic potential (Saha and Ray, 1998; Ghosh et al., 2010).

A positive correlation between growth, FCR, and blood parameters was observed in our results. Efficient metabolism and physical activity are related to a high RBC count. Protease breaks down the indigestible protein into amino acids for better utilization and improved FCR. Digestive capacity, oxygen availability, and metabolic potential collectively determine the synthesis of tissue proteins, which is ultimately related to growth (Blier et al., 1997). Protease activity showed a positive correlation with growth parameters, except for FCE and protein percentage in the carcass. It showed that the enzyme is a secondary metabolite helping in the survival of the cell and may not be necessary to add protein content. As the feed was composed of many carbohydrates, we found a positive relation between amylase and FCR. A positive correlation between cellulase and protein and cellulase and fat suggests that the feed contains sufficient substrates for the enzyme that are converted to protein content, and some intermediates that enter into the fatty acid cycle also help in increasing fat content, and cellulase is effectively utilized. Similarly, growth is not only associated with protein digestion efficiency but also with muscle (filet) and gamete qualities. Thus, the increase in the FCR and survival rate can be due to the presence of essential amino acids produced by digestive enzymes (Supplementary Tables S4A, B).

The positive relationship between diet and gut microbial diversity illustrates the influential impact of diet type on the gut microbiome (Bolnick et al., 2014; David et al., 2014; Delsuc et al., 2014). Probiotic-supplemented diet is one of the potential approaches to alter the microbiology of the fish gut (Standen et al., 2013; Llewellyn et al., 2014; Goncalves and Gallardo-Escarate, 2017). In the current study, *Labeo rohita* fingerlings obtained from a fish hatchery were used, so initial microbiota composition was not analyzed. The present research focused to evaluate the modulation in gut microbial patterns in response to the probiotic-supplemented diet both by culturing and metagenomics approach. Aerobic conditions in culturing also permit the growth of micro-aerophilic and facultative anaerobes along with aerobic bacteria, which support the higher aerobic count in treated groups than in the control by culturing. Herein, we observed a decline in *Enterobacteriaceae* count in probiotic-fed groups, which might be related to competitive exclusion for space, nutrients, and production of anti-pathogenic compounds (Ghadban et al., 1998; Schmitt and Breinig, 2002; Crespo Ramirez, 2016; Zorriehzahra et al., 2016). Previously reported studies also confirmed that the co-culture of yeast and bacteria in many fishes significantly reduced coliforms when compared to fishes fed on a basal diet (Lara-Flores et al., 2003; Marzouk et al., 2008).

Yeast count was higher in treated groups, and Tgc (*G. candidum*) showed the highest count (8.41× 10^7^) than the control fed group with a basal diet. The higher number of *Geotrichum* reads was also observed in Tgc and Tmc groups by metagenomics, which suggests that *G. candidum* adheres well in the community and alone, grows, multiplies, and establishes when incorporated as a probiotic at regular intervals for a specified time, and the same results were reported by Ringo and Gatesoupe (1998), Kiely and Olson (2000), and Syal and Vohra (2014). In comparison, no OTU of *B. cereus* was observed in the Tbc group, which showed that *B. cereus* did not adhere and survived well in *L. rohita* gut. *Enterococcus* count was higher in Tc (2.87 × 10^8^) than in treated groups, which was 5.82 × 10^7^, 6.09 × 10^7^, and 9.59 × 10^7^, respectively. The low count of lactic acid bacteria both in the culturing method and metagenomics approach might be due to improper growth media or conditions for these communities; furthermore, our results were consistent with those of a study that reported that lactic acid bacteria are not the dominant gut community in fish (Ringo and Gatesoupe, 1998).

The metagenomics analysis showed variation in the gut microbial community at each taxonomic level, but the most significant alteration was observed at the species level. Metagenomics analysis showed that the incorporation of *G. candidum* and *B. cereus* causes a reduction in intestinal microbial communities in terms of count while increasing the diversity demonstrated by the number of OTUs and Shannon index. *G. candidum-*treated groups showed significant differences in gut microbial patterns even though they share the same environmental conditions but are fed on a different diet. This trend might be due to the fact that this mixture favors the growth of certain microorganisms and thus decreases richness, while increasing diversity and indicating the gut-modulating effect of probiotics. It is a fact that more than 50% of the variation in gut microbiota is diet-related. Such a decrease in microbial gut community count was observed by Geraylou et al.,(2013) who supplied *L. lactis spp*. lactis ST G45 and AXOS and *B. circulans* ST M53 and AXOS in combination with feed-in *Siberian sturgeon* (Geraylou et al., 2013).

*Proteobacteria* was found to be dominant in all groups. In Tc (control) and Tbc (*B. cereus*), γ class, while in Tgc (*G. candidum*) and Tmc (*B. cereus and G. candidum*), β class of *Proteobacteria* were dominated, which represents that they are core microbial communities. It has been reported earlier that the *Proteobacteria* was also found dominant in the intestine of carp (Desai et al*.*, 2012; Wu et al., 2012). *Bacteroidetes* are observed in all the four treatments used in the trial, which are involved in fermentative metabolism and degradation of oligosaccharides of plant material. This phylum is retrieved mostly from the intestines of different species of carp and is thus helpful in utilizing plant material used in fish feed (Li et al., 2015). A metagenomics study conducted earlier proves the consistent presence of *Bacteroidetes*, *Proteobacteria*, *Firmicutes*, and *Actinobacteria*, although their relative abundance can vary (Gómez and Balcázar, 2008; Ingerslev et al., 2014). *Actinobacteria* are known secondary metabolite producers, especially antibiotics which might be effective in controlling pathogens (Ventura et al., 2007). The maximum percentage of *Actinobacteria* is expressed in Tbc (*B. cereus*), suggesting that *B. cereus* along with *Actinobacteria* phyla showed a strong anti-pathogenic potential.

Here, we observe that the bacterial community was modulated significantly by applied probiotics such as *Pseudomonas* genus and reduced manifold such as *Pseudomonas psychrophilla* and *Pseudomonas syringae* (potent pathogens) in *G. candidum-*treated samples as compared to control. It could be concluded on the basis of previous studies that the probiotics protected the fish by inhibiting pathogens (El-Boshy et al., 2010; Giatsis et al., 2016). It was observed in the present study that *Staphylococcus saprophyticus* was completely phased out by Tgc, giving 0%, which showed that Tgc has modulated the composition and level of indigenous gut community, and its percentage was also reduced in other treatments such as Tbc and Tmc, 0.0188% and 0.0150%, respectively, than in control which showed 0.15%.

The comparatively higher count of *Achrombacter* and *Klebsiella oxytoca*, members of β *Proteobacteria*, commonly present in aquatic habitats, was observed in the Tgc and Tmc groups. *Achrombacter* detoxifies nitrogenous pollutants, thus reducing ammonia stress and keeping fish healthy. Second, it serves to enhance the growth of fishes through tannase production which improves digestibility (Aguilar-Zarate et al., 2014). *Klebsiella oxytoca* possesses a higher cellulolytic activity and can degrade both soluble and non-soluble cellulose and other polysaccharides efficiently (Dantur et al., 2015).

Yeasts though comprise less than one percent of intestinal flora but are inevitable for normal fish gut physiology. (Cahill et al., 1999). Our metagenomics analysis revealed that the opportunistic microbiota was either phased out (*Sporobolomyces lactosus*) or reduced (*Trichosporon* and *Cryptococcus*) in treated groups, highlighting the modulatory properties of applied probiotics. In both probiotic-treated and control groups fed with basal diet, *Ascomycota*, phylum class *Saccharomyces*, was dominated. The genus *Debaryomyces* dominantly recovered from both Tc and Tbc, which demonstrates that feeding of *B. cereus* in a single form did not alter the gut microbiology profile significantly, which is clear from the heat map analysis in which Tc and Tbc clustered together. The same trend was observed in bacterial communities: Tbc and control clustered together. The dominant genus in Tmc and Tgc was *Galactomyces*, and overall yeast and fungal profiles of the gut were altered by feeding *G. candidum* and a mixture of *G. candidum* and *B. cereus.* This indicates that *G. candidum* successfully adhered and colonized in the fish gut tract, as indicated by high reads retrieved from metagenomics analysis. The better adherence of *G. candidum* has been reported previously (Kiely and Olson (2000); Syal and Vohra, 2014). It is suggested that polyamines produced by certain yeasts facilitate their adherence to the mucous epithelium and later on in their colonization (Andlid et al., 1995). *Debaryomyces hansenii* that was dominant in the Tc and Tbc groups is known as killer yeast in literature, which produces toxins and phases out the other yeast, which is also clear from the results as it prevailed more than 80%, but in Tmc and Tgc, *Geotrichum candidum* was dominant, depicting that it competes with *Debaryomyces* and colonizes in the fish gut (Essa et al., 2011; Crespo Ramirez, 2016).

**FIGURE 5:**
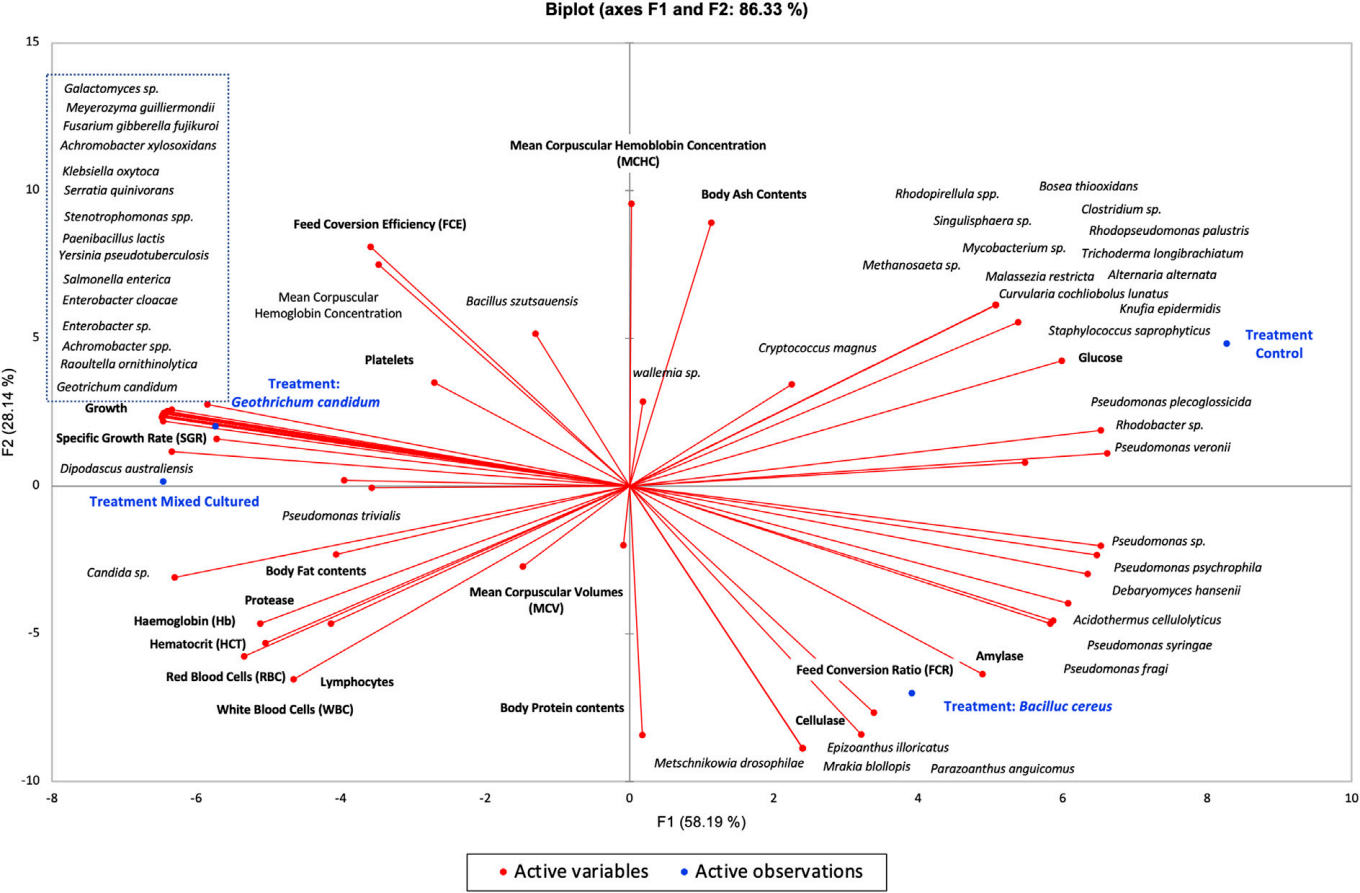
Bi-plot between growth, physiochemistry, hematology, intestinal enzymes and gut microbiota of all treatments Labeo rohita by using Principal component analysis (PCA).

The principal component analysis showed that the Tgc-fed group (*G. candidum* QAUGC01) showed a significant positive correlation among specific growth rate, protein content, protease, and hematocrit. All these factors are interlinked, contributing to the overall increase in metabolism and digestion, thus enhancing growth and activity of fishes. This probiotic has modulated the gut microflora in terms of the absence of potential pathogens of fish such as *Mycobacterium* and *Staphylococcus spp*. as compared to the control-fed group. It might be due to the exclusion principle or due to the production of antimicrobial compounds by *G. candidum* QAUGC01 (Naz et al., 2013; Mukherjee and Ghosh, 2016). The mechanism of modulation needs to be further explored. Moreover, it has enhanced the level of the beneficial bacteria such as *Enterococcus*, *Bacillus*, *Achromobacter*, and *Paenibacillus*; thus, modulation might be a driving force and connectivity toward better physiological parameters. This probiotic supplementation has promoted the health of the fish by modulating the gut microflora. Our results are strongly supported by previous research based on the modulation of gut microbiota by supplementation of yeast extract, which resulted in better growth and health of fish by increasing the relative abundance of beneficial bacteria (Liu et al., 2018). There was a significant correlation between the control group and glucose levels, while all the probiotic-based treatments showed less glucose levels. The probiotic treatment Tmc (*G. candidum* QAUGC01) co-culture with *B. cereus* QAUBC02 showed a significant positive correlation with RBCs, HGB, and WBCs. The improved physiology might be the outcome of an increase in the relative abundance of beneficial microbes and their interaction with the host. Commercial probiotics showed a relatively high number of pathogens such as *Salmonella enterica*, *Klebsiella oxytoca*, and *Serratia quinivorans*. *G. candidum* QAUGC01 has enhanced growth both in single form and co-culture form. *G. candidum* QAUGC01 has executed synergistic effects on *B. cereus* QAUBC02. This combination had significant improvement in fish physiology which might be due to a shift in microbial population toward a balanced microflora. The low percentage of *Bacillus* in Tmc (*G. candidum* QAUGC01 co-culture with *B. cereus* QAUBC02) treatments might be due to numerous environmental and host-related factors. The interactions between microbes remain complex and need further evaluation by using more molecular analysis of metabolic pathways activated by probiotics (Figure 5).

Based on our results, it can be concluded that the combination of *G. candidum* and *Bacillus cereus* enhanced growth, survival, body composition, blood biochemistry, digestive enzymes, and resistance to infections in the rearing of fingerlings. These results revealed that *B. cereus* and *G. candidum* can be used as probiotics both in single and consortium forms. These micro diets based on the co-culture of bacterial and yeast strains could be an attractive, safe, environmentally friendly strategy for sustainable aquaculture by replacing the contemporary control measures. However, selected probiotics’ effects on immune parameters and functional metagenomics of gut modulation need further research.

## Data Availability

The datasets presented in this study can be found in online repositories. The names of the repository/repositories and accession number(s) can be found below: https://www.ncbi.nlm.nih.gov/, *Geotrichum candidum* (QAUGC01, NCBI accession number: KT280407), and *Bacillus cereus* (QAUBC02, NCBI accession number: KY450763).
