# Supplementary material for: Gut microbiome modulation mediated by probiotics: Positive impact on growth and health status of Labeo rohita
Source: Front Physiol. 2022 Sep 9;13:949559. doi: 10.3389/fphys.2022.949559 (PMC9507060; doi:10.3389/fphys.2022.949559)
Supplement: Supplementary file 1 [file Table1.DOCX]

**Table S1: Cumulative table showing invitro characterization of strains (single/ coculture) as potential probiotic candidates**

| **Sr.No.** | **Strains (single/coculture)** | **Percentage Hydrophobicity** | **Growth at pH2** | **Growth at pH 5** | **Growth at pH7** | **Growth at pH 9** | **% survival after 2 hours** | **% survival after 6 hours** | **% survival after24 hours** |
| --- | --- | --- | --- | --- | --- | --- | --- | --- | --- |
| 1. | *B. cereus* QAUBC02 | 30.89^b^(±2.52) | 0.15^i^(±0.001) | 0.35^g^(±0.002) | 0.16^l^(±0.0020 | 0.25^h^(±0.08) | 91.47^a^(±1.61) | 87.31^a^(±1.27) | 85.01^ab^(±1.85) |
| 2. | *G. candidum* QAUGC01 | 21.96^bc^(±1.78) | 0.06^m^(±0.000) | 0.21^hi^(±0.003) | 0.09^l^(±0.00) | 0.28^h^(±0.04) | 38.66^efg^(±0.57) | 33.39^cd^(±1.62) | 34.73^ef^(±2.18) |
| 3. | Co-culture of *G. candidum* QAUGC01and *B. cereus* QAUBC02 | 11.00^de^(±1.74) | 0.101^j^(±0.01) | 0.14^hi^(±0.03) | 0.13^jk^(±0.001) | 0.20^i^(±0.36) | 83.59^a^(±4.97) | 88.21^a^(±1.49) | 79.10^abc^(±2.13) |

†**QAUEH01, *B. cereus G. candidum* QAUGC01, Co-culture *G. candidum* QAUGC01 and *B. cereus* QAUBC02**

‡ Hydrophobicity (%) = [(A0 – A1) / A0] x 100

Where,

‡‡A0= Optical density before mixing the xylene

‡‡ A1= Optical density of aqueous layer

- § %Survival = [OD of bile media / OD of control media] x 100

| - **AAntibiotics** | ***B. cereus*** |
| --- | --- |
|  | **QAUBC02** |
|  |  |
|  |  |
| **Vancomycin** | S |
|  |  |
| **Cefpirome** | R |
|  |  |
| **Ampicillin** | R |
|  |  |
| **Gentamycin** | S |
|  |  |
| **Ceftriaxone** | S |
|  |  |
| **Ciprofloxacin** | S |
|  |  |
| **Chloramphenicol** | S |
|  |  |
| **Ceftazidime** | S |
|  |  |
| **Piperacillin** | R |
|  |  |
| **Moxifloxacin** | I |
|  |  |

**Table S2: Antibiotic sensitivity pattern of bacterial strain.**

†**S: Sensitive I: Intermediate R: Resistant**

**Table S3: Anti-pathogenic activity of the selected isolates and coculture**

| **Isolates** | ***P*. *aeruginosa*** | ***S. enterica*** | ***E. coli*** | ***L. monocytogenes*** | ***S. aureus*** |
| --- | --- | --- | --- | --- | --- |
|  |  |  |  |  | **ATCC** |
| **(Single/co-culture)** | **ATCC 27853** | **ATCC14028** | **ATCC25922** | **ATCC49594** |  |
|  |  |  |  |  | **2593** |
|  |  |  |  |  |  |
|  |  |  |  |  |  |
| ***B. cereus* QAUBC02** | ND* | ND | ND | 28 | 20 |
| ***G. candidum* QAUGC01** | 25.67 | 29 | 28.6 | 25.6 | 30.66 |
| ***G. candidum* QAUGC01** |  |  |  |  |  |
| **co-culture with *B. cereus*** | 26.6 | 24.6 | 20.6 | 21.6 | 26.6 |
| **QAUBC02 combination** |  |  |  |  |  |

†***ND: not determined***

**Table S4a and S4b (45^th^ day and 90^th^ day)**: **Pearson Correlation among different physiological parameters undertaken in study at significance level P<0.05*.* Bold figures represent significant correlation.**

**WBCs= White blood cells; RBCs= Red blood cells;HGB= Hemoglobin;HCT= Hematocrit count;MCV= Mean corpuscular volume; MCH= Mean corpuscular hemoglobin; MCHC= Mean corpuscular hemoglobin concentration;PLT= Platelets;LYM= Lymphocytes;SGR= Specific growth rate; FCR= Feed conversion ratio; FCE= Feed conversion efficiency.**

|  | **Variables** | **1** | **2** | | **3** | **4** | **5** | **6** | **7** |  | **8** |  | **9** | **10** | **11** | **12** | **13** | **14** | **15** |
| --- | --- | --- | --- | --- | --- | --- | --- | --- | --- | --- | --- | --- | --- | --- | --- | --- | --- | --- | --- |
|  |  |  |  |  |  |  |  |  |  |  |  |  |  |  |  |  |  |  |  |
| **1** | WBCs | - |  |  | **.47** | **.75** | .33 | 0.00 | 0.25 | 0.06 |  | **0.65** | 0.01 | **0.54** | **0.51** | -0.04 | 0.07 | **-0.41** | -0.09 |
|  | × 10^3^/µl |  |  |  |  |  |  |  |  |  |  |  |  |  |  |  |  |  |  |
|  |  |  |  |  |  |  |  |  |  |  |  |  |  |  |  |  |  |  |  |
| **2** | RBCs × 10^6^/µl |  |  |  |  | **.55** | **.75** | 0.23 | **-.54** | **-.47** |  | -0.14 | .23 | .25 | .25 | -0.23 | 0.37 | -0.14 | 0.21 |
|  |  |  |  |  |  |  |  |  |  |  |  |  |  |  |  |  |  |  |  |
| **3** | HGB g/dl |  |  |  |  |  | .31 | -.16 | 0.15 | .12 |  | .32 | -.22 | **.57** | **.55** | -0.32 | **0.39** | -0.28 | -.15 |
|  |  |  |  |  |  |  |  |  |  |  |  |  |  |  |  |  |  |  |  |
| **4** | HCT% |  |  |  |  |  |  | **.77** | **-.69** | **-.83** |  | -.32 | .28 | -.21 | -0.22 | 0.18 | -.11 | -.14 | 0.66 |
|  |  |  |  |  |  |  |  |  |  |  |  |  |  |  |  |  |  |  |  |
| **5** | MCVfl |  |  |  |  |  |  |  | **-.56** | **-.87** |  | **-.40** | 0.25 | **-.67** | **-.68** | **0.54** | **-.48** | -0.07 | **.72** |
|  |  |  |  |  |  |  |  |  |  |  |  |  |  |  |  |  |  |  |  |
| **6** | MCH pg |  |  |  |  |  |  |  |  | **.86** |  | **0.52** | **-0.39** | .32 | .30 | -0.19 | .15 | -.25 | **-.53** |
|  |  |  |  |  |  |  |  |  |  |  |  |  |  |  |  |  |  |  |  |
| **7** | MCHC g/dl |  |  |  |  |  |  |  |  |  |  | **.45** | -.36 | **.53** | **.53** | **-.39** | .34 | -0.09 | **-0.61** |
|  |  |  |  |  |  |  |  |  |  |  |  |  |  |  |  |  |  |  |  |
| **8** | PLT×103/µl |  |  |  |  |  |  |  |  |  |  |  | 0.07 | **0.56** | **0.56** | .10 | -0.13 | -.21 | **-.47** |
|  |  |  |  |  |  |  |  |  |  |  |  |  |  |  |  |  |  |  |  |
| **9** | LYM% |  |  |  |  |  |  |  |  |  |  |  |  | 0.03 | 0.05 | .18 | -.23 | 0.29 | 0.19 |
|  |  |  |  |  |  |  |  |  |  |  |  |  |  |  |  |  |  |  |  |
| **10** | %Growth |  |  |  |  |  |  |  |  |  |  |  |  |  | **.99** | **-.39** | **.38** | 0.15 | **-.47** |
|  |  |  |  |  |  |  |  |  |  |  |  |  |  |  |  |  |  |  |  |
| **11** | SGR |  |  |  |  |  |  |  |  |  |  |  |  |  |  | **-.39** | **0..39** | .15 | **-.47** |
|  |  |  |  |  |  |  |  |  |  |  |  |  |  |  |  |  |  |  |  |
| **12** | FCR |  |  |  |  |  |  |  |  |  |  |  |  |  |  |  | **-.98** | 0.00 | **.48** |
|  |  |  |  |  |  |  |  |  |  |  |  |  |  |  |  |  |  |  |  |
| **13** | FCE |  |  |  |  |  |  |  |  |  |  |  |  |  |  |  |  | -0.04 | **-.42** |
|  |  |  |  |  |  |  |  |  |  |  |  |  |  |  |  |  |  |  |  |
| **14** | Protease |  |  |  |  |  |  |  |  |  |  |  |  |  |  |  |  |  | -.26 |
|  |  |  |  |  |  |  |  |  |  |  |  |  |  |  |  |  |  |  |  |
| **15** | Amylase |  |  |  |  |  |  |  |  |  |  |  |  |  |  |  |  |  | - |
|  |  |  |  |  |  |  |  |  |  |  |  |  |  |  |  |  |  |  |  |

|  | **Variables** | **1** | **2** | **3** | **4** | **5** | **6** | **7** | **8** | **9** | **10** | **11** | **12** | **13** | **14** | **15** |
| --- | --- | --- | --- | --- | --- | --- | --- | --- | --- | --- | --- | --- | --- | --- | --- | --- |
|  |  |  |  |  |  |  |  |  |  |  |  |  |  |  |  |  |
| **1** | WBCs × 10^3^/µl | - | **.91** | **.88** | **.55** | **-.39** | **-.53** | **.40** | **.55** | **.94** | **.44** | **.43** | .11 | -.09 | -.11 | -.14 |
|  |  |  |  |  |  |  |  |  |  |  |  |  |  |  |  |  |
| **2** | RBCs × 10^6^/µl |  |  | **.96** | **.71** | -.20 | **-.44** | .32 | .35 | **.80** | **.55** | **.48** | .09 | -.07 | .16 | -.14 |
|  |  |  |  |  |  |  |  |  |  |  |  |  |  |  |  |  |
| **3** | HGB g/dl |  |  |  | **.73** | -.17 | -.35 | .31 | .33 | **.75** | **.57** | **.51** | .07 | -.06 | .12 | -.20 |
|  |  |  |  |  |  |  |  |  |  |  |  |  |  |  |  |  |
| **4** | HCT% |  |  |  |  | **.50** | -.31 | -.37 | -.29 | **.45** | **.46** | .30 | -.11 | .13 | .15 | -.05 |
|  |  |  |  |  |  |  |  |  |  |  |  |  |  |  |  |  |
| **5** | MCVfl |  |  |  |  |  | .11 | **-.94** | **-.91** | **-.39** | -.03 | -.13 | -.25 | .26 | .14 | .16 |
|  |  |  |  |  |  |  |  |  |  |  |  |  |  |  |  |  |
| **6** | MCH pg |  |  |  |  |  |  | -.03 | -.18 | **-.65** | .15 | .09 | -.36 | .34 | .26 | -.37 |
|  |  |  |  |  |  |  |  |  |  |  |  |  |  |  |  |  |
| **7** | MCHC g/dl |  |  |  |  |  |  |  | **.83** | .33 | .19 | .19 | .25 | -.26 | .08 | -.25 |
|  |  |  |  |  |  |  |  |  |  |  |  |  |  |  |  |  |
| **8** | PLT×103/µl |  |  |  |  |  |  |  |  | **.55** | .15 | .23 | .16 | -.17 | -.26 | -.33 |
|  |  |  |  |  |  |  |  |  |  |  |  |  |  |  |  |  |
| **9** | LYM% |  |  |  |  |  |  |  |  |  | .23 | .28 | .16 | -.14 | -.32 | .00 |
|  |  |  |  |  |  |  |  |  |  |  |  |  |  |  |  |  |
| **10** | %Growth |  |  |  |  |  |  |  |  |  |  | **.69** | **-.47** | **.46** | **.40** | **-.73** |
|  |  |  |  |  |  |  |  |  |  |  |  |  |  |  |  |  |
| **11** | SGR |  |  |  |  |  |  |  |  |  |  |  | -.34 | .34 | .27 | **-.56** |
|  |  |  |  |  |  |  |  |  |  |  |  |  |  |  |  |  |
| **12** | FCR |  |  |  |  |  |  |  |  |  |  |  |  | **.99** | .16 | **.44** |
|  |  |  |  |  |  |  |  |  |  |  |  |  |  |  |  |  |
| **13** | FCE |  |  |  |  |  |  |  |  |  |  |  |  |  | **-.17** | **-.41** |
|  |  |  |  |  |  |  |  |  |  |  |  |  |  |  |  |  |
| **14** | Protease |  |  |  |  |  |  |  |  |  |  |  |  |  |  | -.12 |
|  |  |  |  |  |  |  |  |  |  |  |  |  |  |  |  |  |
| **15** | Amylase |  |  |  |  |  |  |  |  |  |  |  |  |  |  | - |
|  |  |  |  |  |  |  |  |  |  |  |  |  |  |  |  |  |
